# Supplementary figures and images for: E2F transcription factors promote tumorigenicity in pancreatic ductal adenocarcinoma
Source: Cancer Med. 2024 Apr 30;13(9):e7187. doi: 10.1002/cam4.7187 (PMC11058697; doi:10.1002/cam4.7187)

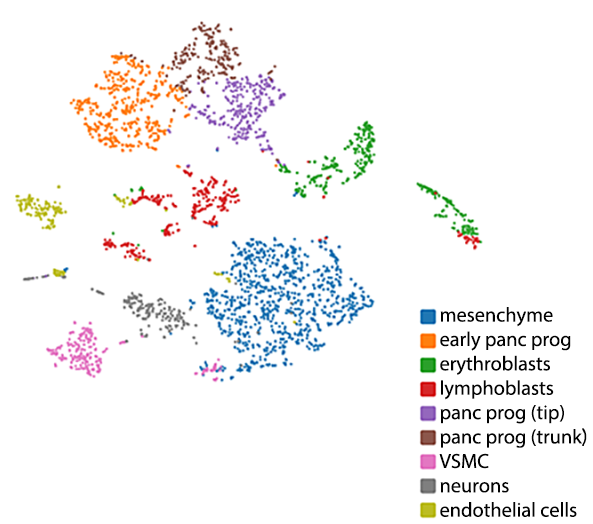

Supplement: Supplementary file 1 — Figure S1. Unbiased identification of cell types from an 8‐week PC embryo. Each color represents a cell cluster from scRNA sequencing revealed by unsupervised clustering and projected on a 2‐D tSNE map. Clusters have been linked to cell types using their transcriptomic signatures (15). [file CAM4-13-e7187-s006.png]

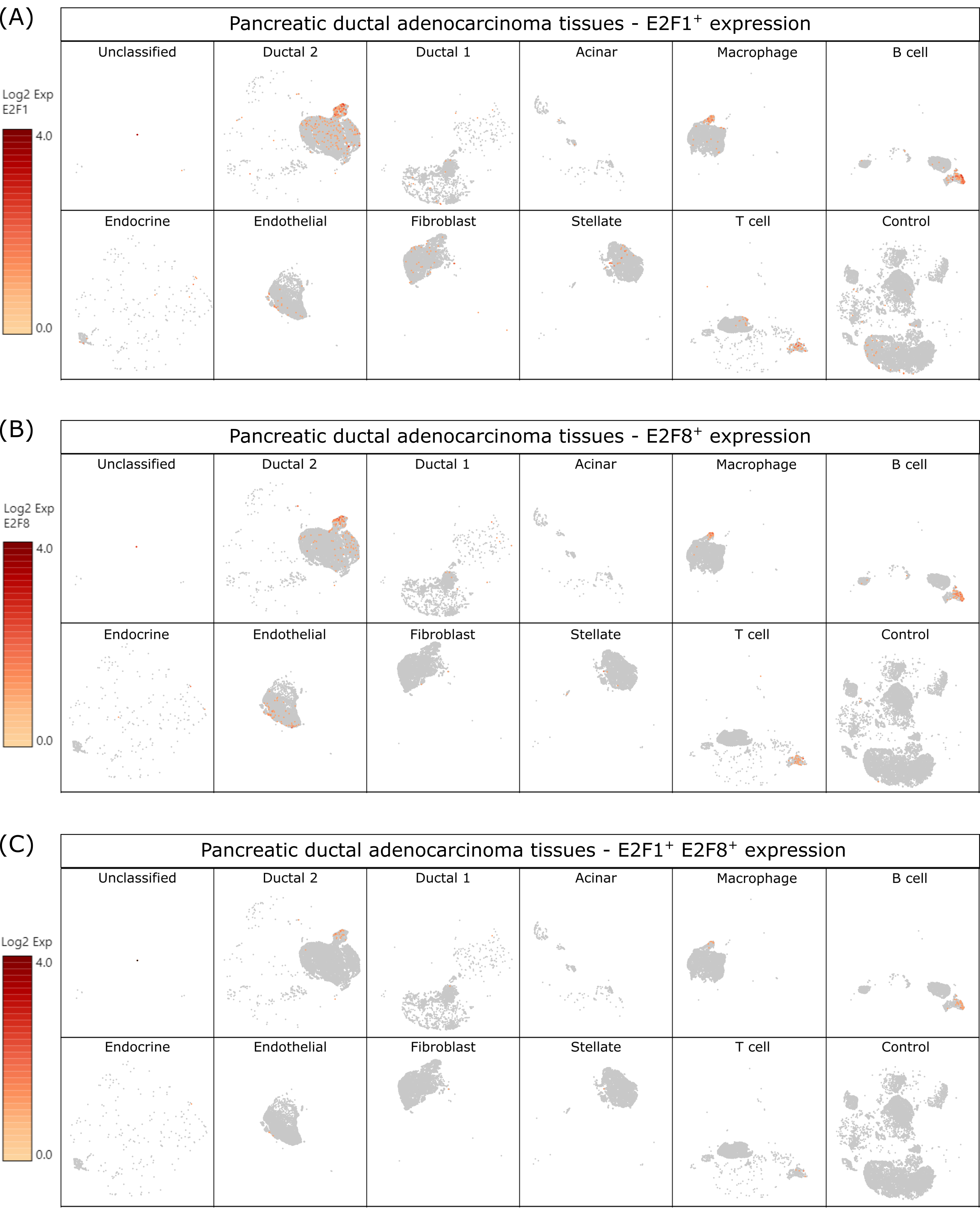

Supplement: Supplementary file 2 — Figure S2. Identification of cell types from PDAC tissues. Localization of E2F1+ (A), E2F8+ (B) and E2F1+E2F8+ (C) cells for each cell type clusters. [file CAM4-13-e7187-s002.png]

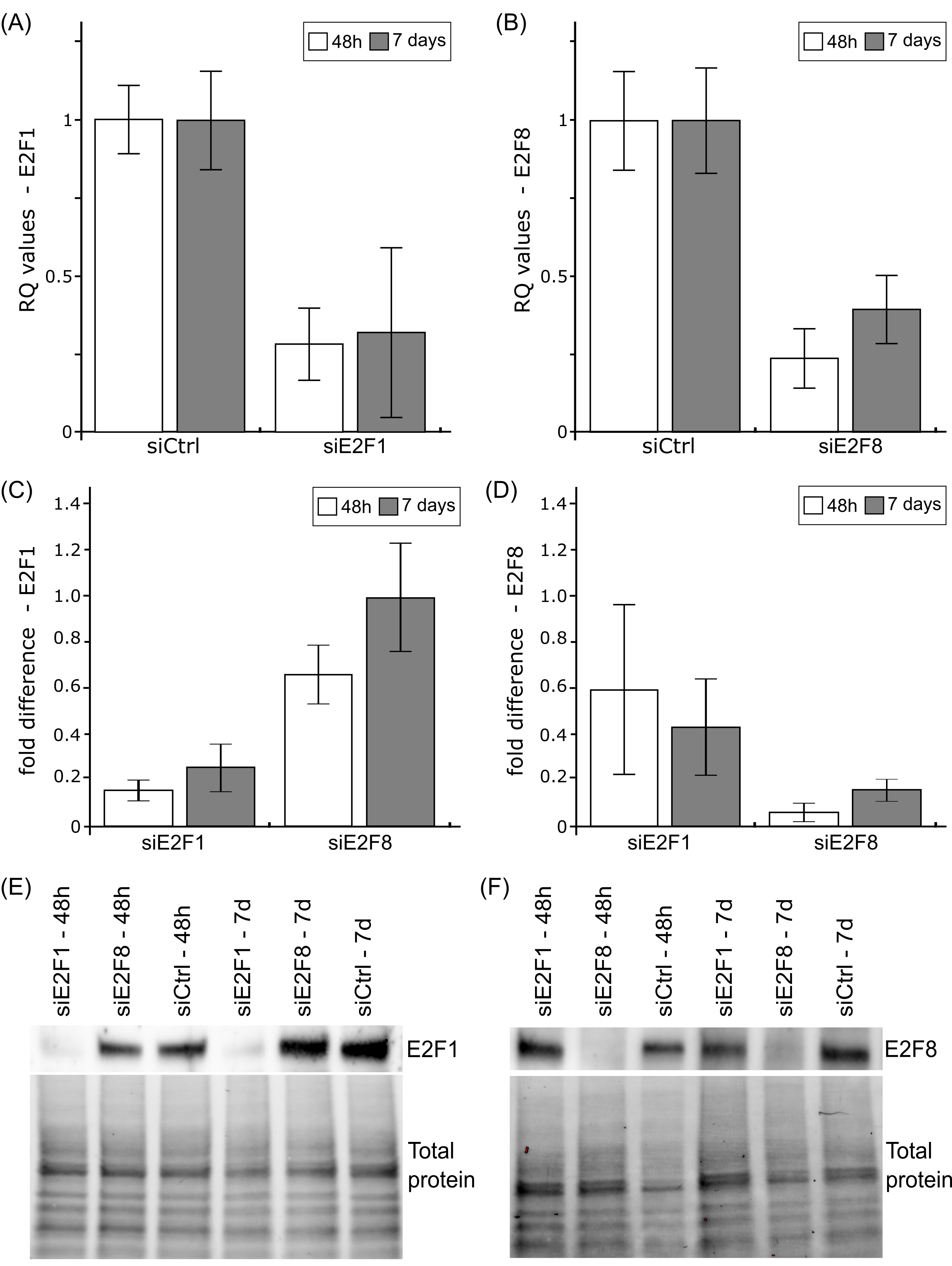

Supplement: Supplementary file 3 — Figure S3. Transfection efficiency determined by RT‐qPCR analyses and Western blot. E2F1 (A) and E2F8 (B) knockdown efficiency 48 h or 7 days after transfection. Expression values were calculated applying the −2∆∆CT algorithm. Estimated relative quantities were normalized for the expression value of the endogenous genes GAPDH, S18 and TBP and calibrated to the negative control samples. E2F1 (C) and E2F8 (D) knockdown efficiency 48 h or 7 days after transfection. E2F1 (E) and E2F8 (F) protein quantifications were normalized for the total amount of protein and calibrated to the negative control samples. [file CAM4-13-e7187-s013.png]

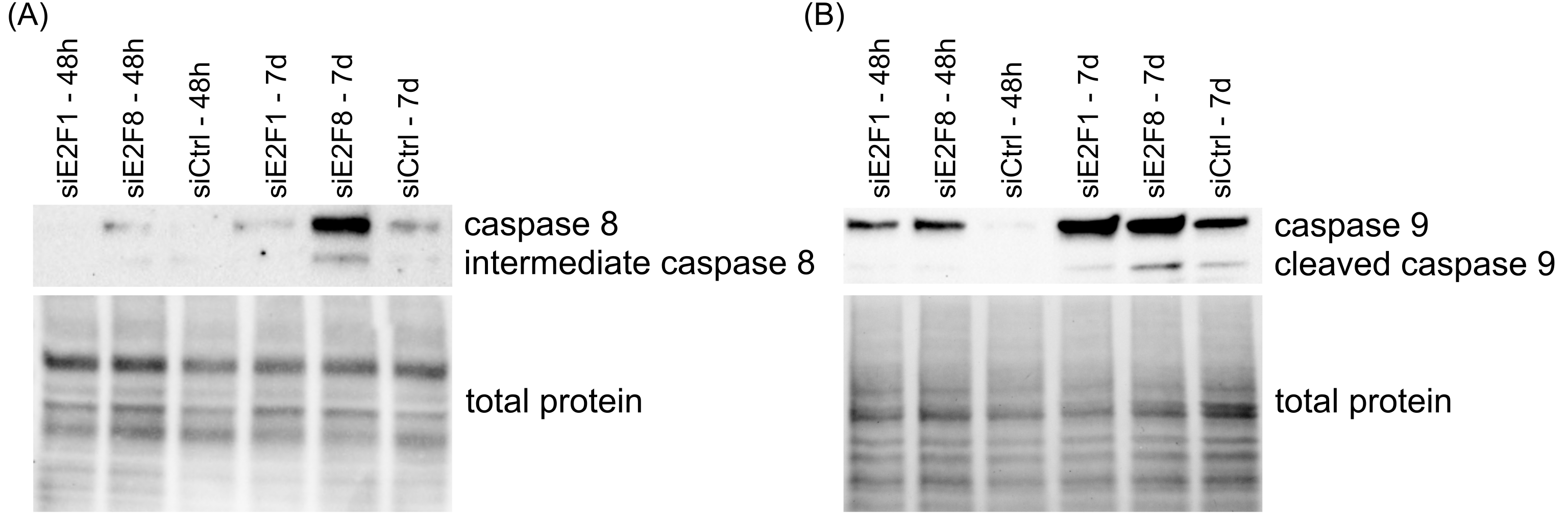

Supplement: Supplementary file 4 — Figure S4. Western blot of caspase 8 and 9. Western blot of Caspase 8 (A) and Caspase 9 (B) and total protein level shown by stain‐free blot staining. [file CAM4-13-e7187-s016.png]
